# Effects of pain, sedation and delirium monitoring on clinical and economic outcome: A retrospective study

**DOI:** 10.1371/journal.pone.0234801

**Published:** 2020-09-02

**Authors:** Marc Deffland, Claudia Spies, Bjoern Weiss, Niklas Keller, Mirjam Jenny, Jochen Kruppa, Felix Balzer

**Affiliations:** 1 Department of Anaesthesiology and Intensive Care Medicine (CCM, CVK), Charité–Universitätsmedizin Berlin, Corporate Member of Freie Universität Berlin, Humboldt Universität zu Berlin and Berlin Institute of Health, Berlin, Germany; 2 Science Communication Unit, Robert Koch Institute, Berlin, Germany; 3 Harding Center for Risk Literacy, Faculty of Health Sciences Brandenburg, University of Potsdam, Potsdam, Germany; 4 Center for Adaptive Rationality, Max Planck Institute for Human Development, Berlin, Germany; 5 Institute of Biometry and Clinical Epidemiology, Charité–Universitätsmedizin Berlin, Corporate Member of Freie Universität Berlin, Humboldt-Universität zu Berlin, and Berlin Institute of Health, Berlin, Germany; 6 Berlin Institute of Health (BIH), Berlin, Germany; University of Colorado Denver, UNITED STATES

## Abstract

**Background:**

Significant improvements in clinical outcome can be achieved by implementing effective strategies to optimise pain management, reduce sedative exposure, and prevent and treat delirium in ICU patients. One important strategy is the monitoring of pain, agitation and delirium (PAD bundle). We hypothesised that there is no sufficient financial benefit to implement a monitoring strategy in a Diagnosis Related Group (DRG)-based reimbursement system, therefore we expected better clinical and decreased economic outcome for monitored patients.

**Methods:**

This is a retrospective observational study using routinely collected data. We used univariate and multiple linear analysis, machine-learning analysis and a novel correlation statistic (maximal information coefficient) to explore the association between monitoring adherence and resulting clinical and economic outcome. For univariate analysis we split patients in an adherence achieved and an adherence non-achieved group.

**Results:**

In total 1,323 adult patients from two campuses of a German tertiary medical centre, who spent at least one day in the ICU between admission and discharge between 1. January 2016 and 31. December 2016. Adherence to PAD monitoring was associated with shorter hospital LoS (e.g. pain monitoring 13 vs. 10 days; p<0.001), ICU LoS, duration of mechanical ventilation shown by univariate analysis. Despite the improved clinical outcome, adherence to PAD elements was associated with a decreased case mix per day and profit per day shown by univariate analysis. Multiple linear analysis did not confirm these results. PAD monitoring is important for clinical as well as economic outcome and predicted case mix better than severity of illness shown by machine learning analysis.

**Conclusion:**

Adherence to PAD bundles is also important for clinical as well as economic outcome. It is associated with improved clinical and worse economic outcome in comparison to non-adherence in univariate analysis but not confirmed by multiple linear analysis.

**Trial registration:**

clinicaltrials.gov NCT02265263, Registered 15 October 2014.

## Introduction

Since the late 1990s, there has been increasing awareness of the impact of clinical management of pain, sedation and delirium in intensive care units (ICUs) [1]. This type of comprehensive management is often referred to as PAD management (pain, agitation/sedation and delirium) and has become an integral part of routine intensive care. There are several reasons why this management is paramount for patients:

Critically ill adults frequently experience pain, which is known to be a major stressor in the ICU and a driver of distress and agitation [2, 3]. Therefore, analgesia is the first priority to minimize sedation. This achieves broad consensus, for example, in the eCASH statement [4].

Deep sedation of ICU patients is associated with adverse outcomes, including longer durations of mechanical ventilation (MV) and increased risk of mortality [5, 6]. Therefore, an approach that minimises sedation is recommended.

Delirium is a common brain dysfunction in critically ill patients and is associated with cognitive dysfunction [7, 8]. Delirium is linked to longer hospital and ICU Length of Stay (LoS) and long-term cognitive impairment a [9]. The development of delirium in ICU patients may be contribute by fluctuations in sedation levels and a non-adequate pain management [10, 11].

Significant improvements in patient short and long term outcome can be achieved by implementing effective strategies to optimise pain management, reduce sedative exposure, and prevent and treat delirium in ICU patients [12, 13]. The most effective measures for implementation are bundles and clinical concepts, such as the ABCDEF approach (**A**ssess, prevent, and manage pain; **B**oth spontaneous awakening and breathing trials: **C**hoice of Analgesia and Sedation; **D**elirium assess, prevent, and manage; **E**arly Mobility and Exercise; **F**amily engagement/empowerment), which aims to improve the management of pain, sedation, and delirium [14].

The implementation of these bundles and especially a diagnostic screening for PAD using validated instruments, which is referred to as PAD monitoring, is recommended in national and international guidelines [15–17].

Although routine monitoring should be part of daily clinical practice, studies have shown low implementation rates [18–21] and demonstrate that implementation of guidelines in clinical practice is a considerable challenge in intensive care [22]. Studies on implementation strategies and barriers currently focus on training, behaviour and organisational structures [23–25]. An additional reason for low implementation rates could be a lack of financial incentivisation of these measures by the reimbursement system. We therefore hypothesise that although adherence to the PAD bundle is linked to improved clinical outcome, there is no sufficient financial benefit to implementing these methods in a Diagnosis Related Group (DRG)-based reimbursement system. This absence of a financial benefit is in turn associated with a worse economic outcome for the hospital, which means lower daily revenues (case mix) and profits.

## Material and methods

The institutional review board (“Ethics Committee of Charité–Universitätsmedizin Berlin”) approved the analysis and waived informed consent (EA 2/092/14). We accessed the date between June 2017 and August 2018. We allocated an alias to data immediately after export. The treatment range was between January 2016 and December 2016. All data come from our hospital.

We set up a retrospective cohort study analysing both clinical and economic outcome. In addition to classical statistical approaches, we used a machine-learning algorithm (Boruta) and the maximal information coefficient (MIC) to analyse the importance (Boruta) and strength (MIC) of effects of monitoring adherence on clinical and economic outcome.

We used routinely collected data and included DRG-invoiced ICU patients who were admitted to and discharged from one eight of the centre’s ICUs in 2016 and who did not receive PAD monitoring as part of a clinical trial. We excluded non adults, re-admissions to more than one ICU ward, patients without a documented day between admission and discharge, deceased patients and cases with no possible CAM-ICU monitoring (see explanation on the main predictor variables).

The study has been registered at ClinicalTrials.gov, number NCT 02265263. The local ethics committee approved the analysis and waived informed consent (EA 2/092/14).

### Data sources

Routine clinical data were acquired from the two electronic patient data management systems used at the hospital (COPRA, Berlin, Germany and SAP, Walldorf, Germany).

### Measures

#### Main predictor variables

The main predictor variables were adherence to pain, sedation and delirium monitoring. Pain, sedation and delirium monitoring were aligned in an algorithm: Starting with sedation monitoring, all patients with a Richmond Agitation Sedation Scale (RASS) of −3 or greater were monitored for delirium with the Confusion Assessment Method for the Intensive Care Unit (CAM-ICU) [26]. Patients with negative CAM-ICU results were assessed for pain using the Visual Agitation Scale (VAS) [27]. In case of a positive CAM-ICU, or if screening for sedation revealed a RASS of −4 or less, patients were screened for pain using the Behavioural Pain Scale (BPS) [28].

The adherence to pain, sedation and delirium monitoring for a patient was calculated as follows: the number of “adhered shifts” was divided by the total number of ICU shifts in which the patient was treated. Shifts on the day of admission and the day of discharge were not considered. Accordingly, the adherence could assume a value between 0 and 100%. A patient's ICU shift was rated as “adhered” if the patient was monitored at least once per shift. Patients with an Adherence of 100% were classified as “achieved” for univariate analysis. If the patient received no delirium monitoring, the shift was only rated as adhered when there was no RASS assessment or CAM-ICU was not possible. Patients for whom CAM-ICU monitoring was not possible were not considered in the analysis of CAM-ICU monitoring adherence. We evaluated pain, sedation and delirium separately, not as bundle.

#### Outcome variables

The clinical outcome variables were hospital LoS, ICU LoS and duration of mechanical ventilation. The economic variables included case mix per day and profit per day because of their incentive effect for hospitals. Profit and turnover or case mix influence the management of a hospital: Profit per day addresses two points, a day view and the difference between turnover and costs. Turnover is a variable concerning the market share of a hospital. Therefore, we use profit per day and not total costs or costs per day.

The case mix (measured in case mix points) was derived from Diagnosis Related Groups (DRG). These points multiplied with a base rate (measured in EUR) are the substantial part of hospital revenue for a hospital case. Profit per day was calculated by case mix multiplicated with baserate plus other receipts minus the case costs documented for the German nationwide institute of hospital revenue and costs calculations (InEK: Institut für das Entgeltsystem im Krankenhaus).

#### Covariates

The control variables for the multiple linear analysis and machine-learning algorithm were determined a priori based on available literature and clinical experience. They included age and the Acute Physiology and Chronic Health Evaluation II (APACHE II) scale score.

### Analysis

We used both a classical statistical approach and a machine-learning analysis approach (the Boruta algorithm with MIC). While the classical approach uses all available data to explain or best describe particular linear associations and correlations, the use of cross-validation via a non-linear machine learning method allowed us to identify robust predictor variables for our outcome measures that are potentially non-linearly related to the latter. Cross-validation is the process of training the model on a subset of the data and then allowing it to assess the remainder of the cases in the dataset. This reduces the chance of model overfitting, e.g., capturing spurious correlations. In short, we supplemented the classical statistical analyses by alternative approaches for the identification of variables that are non-linearly related to our outcome and that are likely to also be predictive in new patient populations, i.e., that are likely to generalise.

For all classical statistical analyses, we used SPSS Version 24.0.0.0 (IBM SPSS Statistics). For univariate analysis, we split patients into groups. To differentiate between the monitored (monitoring adherence achieved) and not-monitored (monitoring adherence not achieved) patients, we set a 100% adherence quote. To differentiate between disease severity we used APACHE < = 10-group, APACHE 11-20-group and APACHE >20-group. For testing the association between monitoring adherence and outcome, we also used linear regression models.

For the machine-learning analysis, we used RStudio version 1.1.419 (R Foundation for Statistical Computing), and the “Boruta” package (for details, see [29]). Boruta is a random-forest-based method of feature selection. A random forest [30] is an ensemble model that constructs a multitude (often thousands) of decision trees based on the data and then makes a committee prediction (e.g., the algorithm predicts whatever the majority of the individual decision trees predict). These models can capture non-linear and non-monotonic relationships between the input variables and outcome criteria that linear models would not be able to capture. The Boruta algorithm builds these random forests from the dataset. It then randomly shuffles the values of each variable one by one to test whether the forest’s classification performance declines when a variable’s potential statistical relationship with the criterion is eliminated by this process of value randomisation. This method is also known as permutation-based variable importance.

The Boruta algorithm only provides the relative strength of association of the different input variables; therefore, the association strength cannot be easily compared across the different outcome criteria. To identify the strength of the associations, we used the maximal information coefficient (MIC) [31], a measure of information entropy that, like random forests, is not limited to specific types of functions (linear, non-linear, or non-monotone). MIC values can range from 0 to 1 and tend to be similar to R^2^ in size and interpretation.

## Results

**Fig 1:**
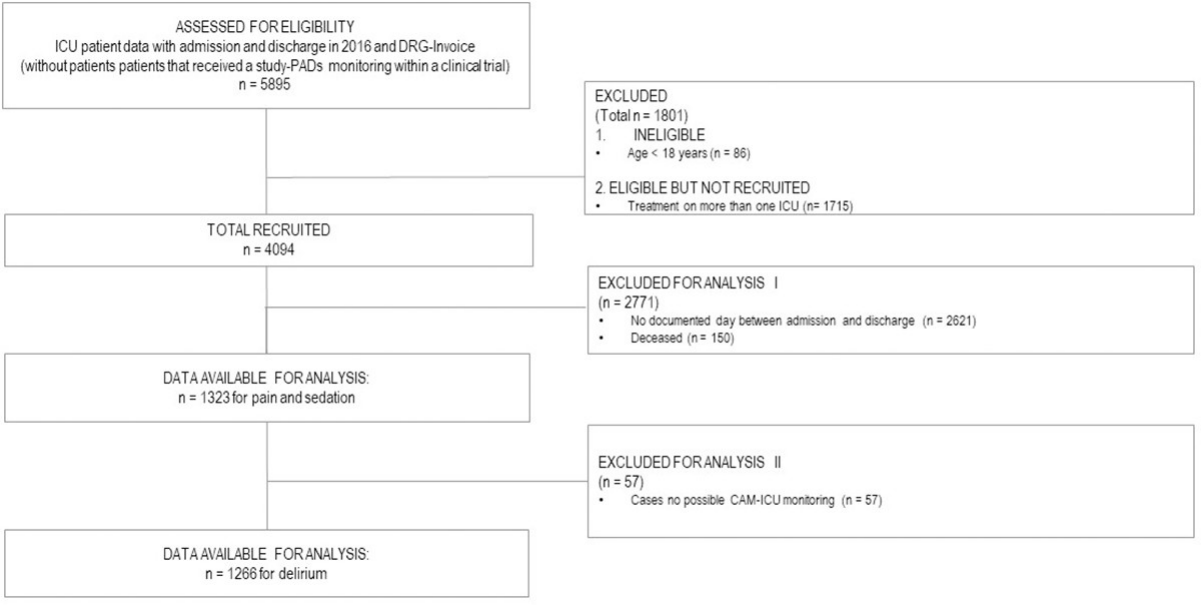
Consort diagram.

We included 1323 patients for pain and agitation/sedation monitoring and 1266 patients for delirium monitoring (Fig 1). The groups differed because some patients had insufficient RASS data: For example, a patient with a RASS of -3 or less during their hospital stay could not be monitored for delirium (see the explanation of the main predictor variables).

The median age of the admitted patients was 68 years, 84.4% received surgery, and 60% were male. The median ICU LoS was 4 days, while the median hospital LoS was 12 days, and the median duration of mechanical ventilation was 32 hours. The median monitoring adherence was 91.7% for pain, 90.4% for agitation/sedation and 100% for delirium (Table 1). The median economic outcomes were 0.44 case mix per day and 11.05 EUR profit per day. The median APACHE II score at admission was 14.

**Table 1:** Patient characteristics.

Characteristics	(n = 1323)
Surgery (%)	84.4
Male (%)	60.0
Age, y [median (Q1 to Q3)]	68(56 to 76)
Hospital LoS, d [median (Q1 to Q3)]	12(7 to 19)
ICU LoS, d [median (Q1 to Q3)]	4(3 to 8)
Duration of MV, h [median (Q1 to Q3)]	32(0.0 to 109)
Pain monitoring adherence,	
% [median (Q1 to Q3)]	91.7(80.9 to 100)
% [adherence achieved]	60.7
Agitation/sedation monitoring adherence,	
% [median (Q1 to Q3)]	90.4(76.2 to 100)
% [adherence achieved]	67.0
Delirium monitoring adherence,	
% [median (Q1 to Q3)]	100(92.3 to 100)
% [adherence achieved]	64.6
Profit per day, EUR [median (Q1 to Q3)]	11.05(-240.6 to 445.3)
Case mix per day, points [median (Q1 to Q3)]	0.44(0.26 to 0.71)
APACHE II score on admission, points [median (Q1 to Q3)]	14(9 to 22)

LoS = length of stay, APACHE II = acute physiology and chronic health evaluation, MV = mechanical ventilation, y = years, h = hours

### Relating monitoring and outcome

#### a) Two-group-analyses

For pain monitoring adherence (Tables 2 and 3), in most cases, we observed statistically significant improvement of clinical outcome and worse economic outcome for all patients and for each disease severity patient group. Exceptions were case mix per day for the APACHE>20 group, profit per day for the APACHE < = 10 group and the APACHE 11–20 group and profit per day for the APACHE>20 group. All exceptions were not significant.

**Table 2:** Relating monitoring and clinical outcome.

			Cases	Hospital LoS		ICU LoS		Duration of MV	
			No.	Med. (Q1 to Q3)	p	Med. (Q1 to Q3)	p	Med. (Q1 to Q3)	p
Pain monitoring adherence	All patients	Not achieved	803	13*(8 to 22)	**<0.001**	6*(4 to 11)	**<0.001**	60*(<0.001to 167)	**<0.001**
		Achieved	520	10*(6 to 15)	**<0.001**	3*(2 to 4)	**<0.001**	0*(<0.001 to 55.75)	**<0.001**
	APACHE < = 10	Not achieved	230	11*(19 to 7)	**0.003**	5*(8 to 3)	**<0.001**	29*(0.001 to 107.75)	**<0.001**
		Achieved	189	9*(6 to 14)	**0.003**	2*(2 to 4)	**<0.001**	0*(<0.001 to 23.50)	**<0.001**
	APACHE 11–20	Not achieved	319	13*(8 to 22)	**<0.001**	6*(3 to 10)	**<0.001**	48*(<0.001 to 134)	**<0.001**
		Achieved	212	10*(7 to 15)	**<0.001**	3*(2 to 4)	**<0.001**	0*(<0.001 to 55)	**<0.001**
	APACHE >20	Not achieved	254	16*(9 to 25)	**<0.001**	7*(5 to 17)	**<0.001**	122,5*(34.75 to 316)	**<0.001**
		Achieved	119	10*(7 to 15)	**<0.001**	4*(3 to 5)	**<0.001**	45*(<0.001 to 86)	**<0.001**
Sedation/agitation monitoring adherence"	All patients	Not achieved	886	13*(8 to 21)	**<0.001**	5*(3 to 10)	**<0.001**	51*(<0.001 to 149)	**<0.001**
		Achieved	437	10*(6 to 15.5)	**<0.001**	3*(2 to 4)	**<0.001**	0*(0 to 59)	**<0.001**
	APACHE < = 10	Not achieved	270	10(6 to 18)	0.161	4(2 to 8)	<0.001	3*(<0.001 to 92.75)	<0.001
		Achieved	149	10(6 to 14)	0.161	3(2 to 4)	<0.001	<0.001*(<0.001 to 29)	<0.001
	APACHE 11–20	Not achieved	349	13*(8 to 21)	**<0.001**	5*(3 to 9)	**<0.001**	39*(<0.001 to 123)	**<0.001**
		Achieved	182	10*(7 to 17.25)	**<0.001**	3*(2 to 4)	**<0.001**	<0.001*(0.001 to 60.25)	**<0.001**
	APACHE >20	Not achieved	267	16*(10 to 25)	**<0.001**	7*(5 to 16)	**<0.001**	110*(38 to 302)	**<0.001**
		Achieved	106	9.5*(6.75 to 14.25)	**<0.001**	4*(2.75 to 6)	**<0.001**	34*(<0.001 to 86)	**<0.001**
Delirium monitoring adherence	All patients	Not achieved	448	15*(8 to 24)	**<0.001**	7*(4 to 13.75)	**<0.001**	85*(8 to 222)	**<0.001**
		Achieved	818	11*(7 to 17)	**<0.001**	4*(2 to 6)	**<0.001**	16*(<0.001 to 76)	**<0.001**
	APACHE < = 10	Not achieved	116	11(6 to 21)	0.050	6*(3 to 11)	**<0.001**	54,5*(<0.001 to 146.75)	**<0.001**
		Achieved	278	10(6 to 15)	0.050	3*(2 to 5)	**<0.001**	<0.001*(<0.001 to 49.25)	**<0.001**
	APACHE 11–20	Not achieved	183	14*(8 to 23)	**0.001**	6*(4 to 12)	**<0.001**	69*(<0.001 to 176)	**<0.001**
		Achieved	326	11*(8 to 17)	0.001	4*(2 to 6)	**<0.001**	11*(<0.001 to 69.5)	**<0.001**
	APACHE >20	Not achieved	149	18*(10 to 27.5)	<0.001	9*(6 to 20)	**<0.001**	149*(59 to 444.5)	**<0.001**
		Achieved	214	12*(7–17.25)	<0.001	5*(3–7)	**<0.001**	51*(1–112.75)	**<0.001**

* = p<0.05 (Mann-Whitney-U-Test), LoS = length of stay, APACHE II = acute physiology and chronic health evaluation, MV = mechanical ventilation, Med. = median / (quartile 1 to quartile 3)

**Table 3:** Relating monitoring and economic outcome.

			Cases	Case mix per day		Profit per day	
			No.	Med. (Q1 to Q3)	p	Med. (Q1 to Q3)	p
Pain monitoring adherence	All patients	Not achieved	803	0.48*(0.29 to 0.73)	**<0.001**	27.40*(-223.61 to 496.30)	**0.015**
		Achieved	520	0.39*(0.23 to 0.64)	**<0.001**	-33.08*(-270.44 to 370.62)	**0.015**
	APACHE < = 10	Not achieved	230	0.43*(0.25 to 0,70)	**0.005**	-21.12(-286.75 to 511.47)	0.055
		Achieved	189	0.32*(0.21 to 0.60)	**0.005**	-113.22(-280.76 to 205.22)	0.055
	APACHE 11–20	Not achieved	319	0.45*(0.265 to 0.70	**0.038**	-9.75(-269.19 to 445.29)	0.469
		Achieved	212	0.36*(0.23 to 0.64)	**0.038**	-28.31(-274.76 to 419.81)	0.469
	APACHE >20	Not achieved	254	0.55(0.36 to 0.80)	0.102	124.70(-138.42 to 615.73)	0.679
		Achieved	119	0.49(0.31 to 0.71)	0.102	137.73(-207.29 to 532.74)	0.679
Sedation/agitation monitoring adherence"	All patients	Not achieved	886	0.49*(0.29 to 0.77)	**<0.001**	50.33*(-217.26 to 534.16)	**<0.001**
		Achieved	437	0.34*(0.22 to 0.61)	**<0.001**	-71.39*(-278.35 to 302.10)	**<0.001**
	APACHE < = 10	Not achieved	270	0.44*(0.26 to 0.73)	**<0.001**	26.11*(-252.61 to 586.55)	**<0.001**
		Achieved	149	0.30*(0.19 to 0.51)	**<0.001**	-144.31*(-344.96 to 74.24)	**<0.001**
	APACHE 11–20	Not achieved	349	0.47*(0.26 to 0.73)	**<0.001**	8.72(-243.62 to 459.53)	0.134
		Achieved	182	0.34*(0.21 to 0.60)	**<0.001**	-77.43(-286.23 to 327.93)	0.134
	APACHE >20	Not achieved	267	0.57*(0.38 to 0.79)	**0.019**	130.49(-141.11 to 643.58)	0.526
		Achieved	106	0.46*(0.30 to 0.71)	**0.019**	139.49(-180.65 to 503.26)	0.526
Delirium monitoring adherence	All patients	Not achieved	448	0.52*(0.32 to 0.78)	**<0.001**	45.02(-230.23 to 481.08)	0.13
		Achieved	818	0.39*(0.24 to 0.66)	**<0.001**	-9.23(-252.77 to 387)	0.13
	APACHE < = 10	Not achieved	116	0.49*(0.28 to 0.81)	**<0.001**	-45.40(-299.38 to 501.20)	0.325
		Achieved	278	0.33*(0.21 to 0.60)	**<0.001**	-70.95(-303.41 to 280.49)	0.325
	APACHE 11–20	Not achieved	183	0.47*(0.28 to 0.73)	**0.004**	5.24(-251.34 to 477.18)	0.172
		Achieved	326	0.37*(0.22 to 0.62)	**0.004**	-42.63(-277.46 to 350.86)	0.172
	APACHE >20	Not achieved	149	0.55(0.38 to 0.79)	0.196	116.41(-156.88 to 518.23)	0.321
		Achieved	214	0.51(0.32–0.76)	0.196	155.17(-132.74–644.72)	0.321

* = p<0.05 (Mann-Whitney-U-Test), LoS = length of stay, APACHE II = acute physiology and chronic health evaluation, MV = mechanical ventilation, Med. = median / (quartile 1 to quartile 3)

For sedation monitoring adherence (Tables 2 and 3), in most cases, we observed statistically significant improvement of clinical outcome and worse economic outcome for all patients and for each disease severity patient group. Exceptions were hospital LoS for the APACHE < = 10-group, the ICU LoS for the APACHE < = 10 group, profit per day for the APACHE 11–20 groups and profit per day for the APACHE>20 group. All exceptions were not significant.

For delirium monitoring adherence (Tables 2 and 3), in most cases, we observed significantly better clinical outcome and worse economic outcome for all patients and for each disease severity patient group. Exceptions were hospital LoS for the APACHE < = 10 group, case mix per day for the APACHE >20 group, profit per day for the APACHE < = 10 and APACHE 11–20 group and profit per day for the APACHE >20 group. All exceptions were not significant.

#### b) Multiple linear regression

Multiple linear regression found different associations (Table 4). An increase of APACHE II score was associated with an increased hospital LoS. An increase of sedation monitoring adherence and APACHE II score was associated with an increase in days of ICU LoS. An increase of pain monitoring adherence was associated with a decreased ICU LoS. An increase of sedation monitoring adherence and APACHE score was associated with an increase in duration of MV. An increased age was associated with a decreased duration of MV. An increase of pain monitoring adherence, age and APACHE score was associated with an increased case mix per day. An increase in delirium monitoring adherence was associated with a decreased case mix per day. An increase of pain monitoring adherence, age and APACHE score was associated with an increased profit per day. Adjusted R-square was very low for all multiple linear regression.

**Table 4:** Multiple linear regression.

	clinical outcome									economic outcome					
	Hospital LoS (adjusted R-square = .021; F = 6.409; p < .001)			ICU LoS (adjusted R-square = .047; F = 13.413; p < .001)			Duration of MV (adjusted R-square = .059; F = 16.907; p < .001)			Case mix per day (adjusted R-square = .042; F = 12.037; p < .001)			Profit per day (adjusted R-square = .020; F = 6.062; p < .001)		
	*B*	*95% CI*	*p*	*B*	*95% CI*	*p*	*B*	*95% CI*	*p*	*B*	*95% CI*	*p*	*B*	*95% CI*	*p*
pain monitoring adherence	-0,018	-0.041 to 0.005	0,430	**-0,045**	**-0.063 to -0.027**	**0,013**	-0,693	-1.11 to -0.275	0,097	**0,170**	**0.109 to 0.231**	**0,005**	**2,894**	**1.720 to 4.068**	**0,014**
sedation monitoring adherence	0,022	0.002 to 0.042	0,263	**0,049**	**0.033 to 0.065**	**0,002**	**1,011**	**0.642 to 1.38**	**0,006**	-0,103	-0.157 to -0.05	0,055	-0,576	-1.614 to 0.461	0,578
delir monitoring adherence	-0,010	-0.032 to 0.013	0,660	-0,006	-0.023 to 0.012	0,751	-0,598	-1.011 to -0.185	0,148	**-0,194**	**-0.254 to -0.133**	**0,001**	-0,363	-1.525 to 0.799	0,755
APACHE	**0,220**	**0.18 to 0.261**	**0,000**	**0,228**	**0.196 to 0.26**	**0,000**	**6,313**	**5.563 to 7.062**	**0,000**	**0,505**	**0.396 to 0.614**	**0,000**	**8,413**	**6.305 to 10.521**	**0,000**
age	-0,028	-0.051 to -0.005	0,230	-0,014	-0.032 to 0.004	0,443	**-0,955**	**-1.381 to -0.53**	**0,025**	**0,176**	**0.114 to 0.238**	**0,005**	**2,431**	**1.233 to 3.629**	**0,043**

* = p<0.05 (XXX), LoS = length of stay, APACHE II = acute physiology and chronic health evaluation, MV = mechanical ventilation

### Predicting outcome based in monitoring adherence

#### a) Relative predictability

**Fig 2:**
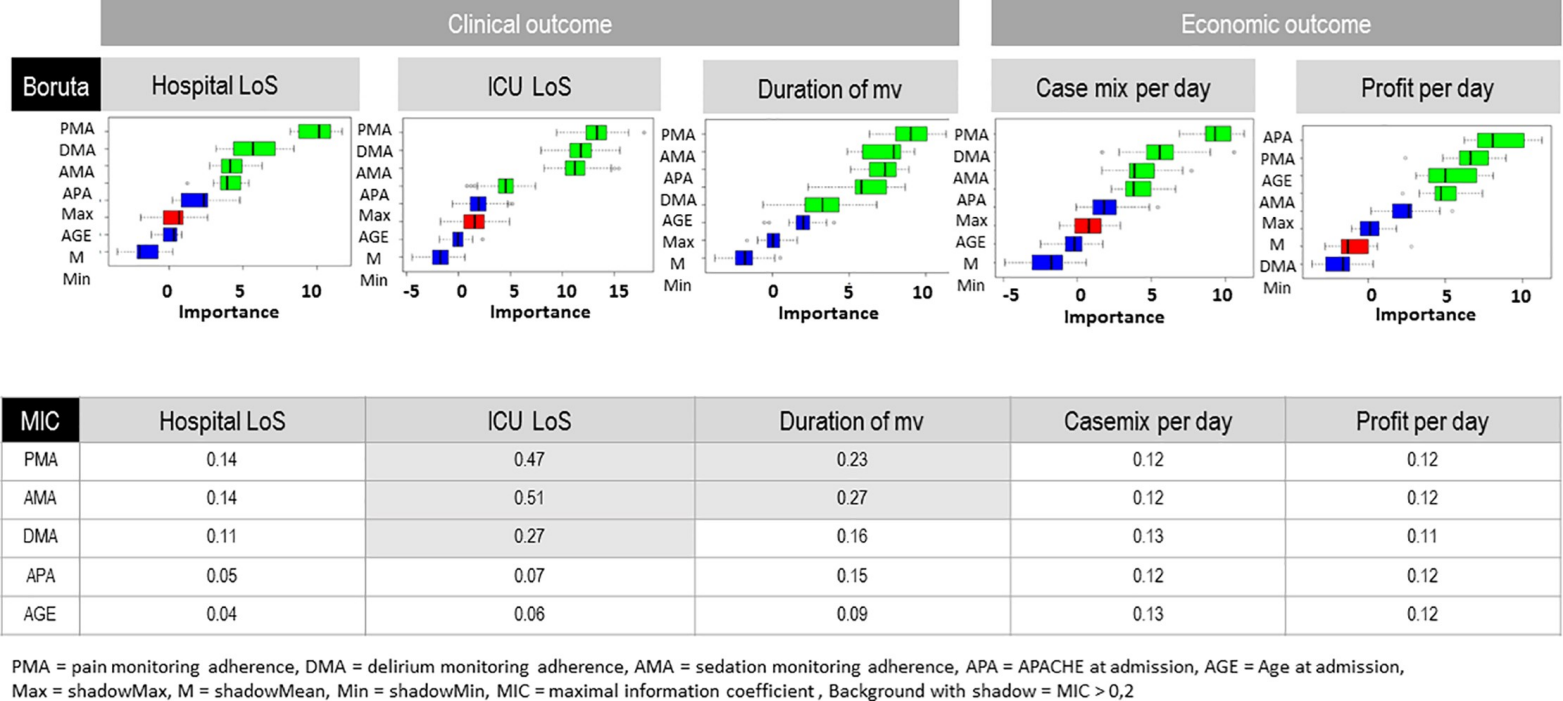
Results of Boruta and MIC for effects of monitoring adherence to clinical and economic outcome. The green predictor variables are deemed predictive by the Boruta algorithm while the red are not.

The machine-learning analysis showed that pain monitoring adherence was the most important predictor of clinical outcome (hospital LoS, ICU LoS, duration of MV) and case mix per day (see Boruta in Fig 2). Furthermore, sedation monitoring was more important to clinical outcome and case mix per day than the APACHE II score. The APACHE II was more important to profit per day than the other variables. However, delirium monitoring was important to clinical and economic outcome and was more important than the APACHE II score for hospital LoS, ICU LoS and case mix per day, but not for duration of MV. Delirium was further not predictive of profit per day. Age was not important for hospital LoS, ICU LoS or case mix per day. Age was important for the duration of MV but was less important than PAD monitoring and APACHE II score. Age was also important for profit per day but less important than the APACHE II and pain monitoring and agitation monitoring.

#### b) Associations between monitoring adherence and outcome

The strongest associations were shown between PAD monitoring and ICU LoS, with MICs of 0.47, 0.51 and 0.27, respectively (see MIC in Fig 2). A strength of association higher than 0.2 was found between pain and sedation management and the duration of MV (see MIC in Fig 2). The strength of the association between pain, sedation and delirium monitoring and hospital LoS were all lower than 0.15. The strength of the association between pain, agitation/sedation and delirium monitoring and case mix per day were all lower than 0.14.

## Discussion

We revealed that adherence to PAD bundles was associated with improved clinical outcome (hospital LoS, ICU LoS, duration of MV) but worse economic outcome (case mix per day, profit per day). Unfortunately, the result could not be confirmed in a multiple linear regression. A cause for the less pronounced effect in our cohort could be the overall high adherence and, thus, a smaller effect size.

In addition to the classical statistical evaluation, we used the machine-learning algorithm Boruta and a novel statistic (MIC). The Boruta algorithm revealed that monitoring adherence was important for clinical and economic outcome. Pain monitoring was the most important predictor of clinical outcome and case mix per day. The analysis showed that sedation and delirium monitoring are less important than pain monitoring but in most cases are more important than APACHE II score and age. While age was irrelevant, Boruta showed that the APACHE II score is an important predictor of clinical and economic outcome. This matches studies showing that the APACHE II score can predict hospital and ICU LoS as well as mortality rates [32].

While the Boruta algorithm only describes the relative strength of the associations of different input variables, MIC analysis identifies the strength of the individual associations. The strongest associations were shown between pain, agitation/sedation and delirium monitoring and ICU LoS.

Our clinical data are in line with most previous studies showing that adherence to the PAD bundle is independently associated with improved clinical outcome. For example, Luetz and colleagues found an independent association between delirium monitoring and in-hospital mortality for ventilated patients, Mansouri et al. found a substantial reduction in the duration of MV, ICU LoS, and mortality through protocol-directed PAD management and Dale et al. found decreases in delirium, duration of MV as well as ICU and hospital LoS [33–35].

Despite the favourable clinical outcome, the economic outcome within the German DRG system was associated with a decrease in both case mix per day and profit per day shown by univariate analysis but not conformed by multiple linear analysis. But it has to be considered, adjusted R-square was very low for all multiple linear regression. The focus of implementation of a DRG system was primarily keeping the quality on the existing level and reining back the costs. The focus on better quality and a change of incentive structure is an actual trend (see below).

The relevance of DRG for Germany is more important than in other health systems: While in other countries, the rate is much lower (40%-50%), in Germany, 80% of hospital finances are covered by DRG [36]. There were no surcharges for fixed costs and quality until 2020, due to a DRG system. Although there are no studies showing false incentives by DRG system, there is a trend concerning the implementation of surcharges for quality and financing of some fixed costs by surcharges. For example, Germany implemented a surcharge for nursing expenses since 2020. A recent study showed that the type and amount of reimbursement has a strong influence on the chosen treatment strategy [37]. This could be a way to optimise incentives in a DRG dominated system.

### Strengths and limitations of this study

Our study has several strengths. This is the first study to use machine-learning analyses to examine the importance of monitoring in terms of both clinical and economic outcome. Furthermore, this study is the first to evaluate the economic effects of all PAD bundle monitoring values in a DRG system by case mix and profit per day.

Our study has several limitations. The first limitation of our results is that we used routine data from our clinical systems. Because of permanent validation procedures, the quality of the data can be assumed to be high, but incorrect entries in individual datasets cannot be excluded.

A further limitation is seen by measuring economic outcome only with turnover (casemix) and profit per day. We did not separately analysed the total costs. With shorter ICU and hospital LoS the total costs are probably lower than with regular LoS. Our economic view based just on the figures with an (economic) incentive for hospital (see explanation on outcome variables).

Additionally, case mix is a highly context-sensitive system. Hence, the results might not be applicable to different international health care systems. Additionally, the German national system is constantly changing, and the results for years other than 2016 could be different.

The economic analysis is only for Germany. A predication for other health systems is not possible because of the different health reimbursement systems. Many countries have a DRG system but using own databases and cost accounting guidelines.

The above cited study regarding APACHE and ICU LoS used the APACHE IV, while our hospital uses the APACHE II [32]. Furthermore, we assume in our economic analysis that reductions in LoS could allow additional patients to be treated. That is the reason for using case mix and profit per day and not per case. Factors other than the PAD bundle certainly also influenced clinical and economic outcome, but these factors could not be quantified.

A further limitation is that we treated anything less than full adherence as non-adherence in univariate analysis. The reason for this choice was the high implementation rates at our hospital. The national guideline recommends that monitoring should occur during a minimum of 70% of all shifts [17]. The 70% threshold is known from other contexts (antibiotics stewardship) to be an effective margin for reaching a significant effect by implementing standard operating procedures [38]. However, greater implementation still has effects, as our clinical data show. Using a lower percentage threshold would decrease the effect size and is therefore the more conservative approach. Our results might be a reason to conduct further studies concerning the need for higher target values.

Adherence to monitoring and analysis of effects depends on the guideline used by the institution because there are often differences in the details of different guidelines; e.g., the Spanish guideline requires sedation monitoring every six hours only for mechanically ventilated patients and gives a target value of 95% [39]. The Germany national guideline requires sedation monitoring every eight hours for every patient and gives a standard of 70% [17]. However, independent of the individual recommendations of a guideline, all advice concerning PAD management increases the sensitivity of employees in intensive care and helps to increase quality of care.

There is a need to conduct prospective studies on the topic of PAD monitoring adherence to validate our results and focus more closely on economic outcome to improve incentives for quality in a DRG-based system. Further studies should also aim at cohorts with larger differences regarding PADs adherence for confirmatory analysis.

## Conclusion

Adherence to PAD bundle is important for clinical as well as economic outcome. It is associated with improved clinical and worse economic outcome in comparison to non-adherence in univariate analysis but not confirmed by multiple linear analysis.

## Supporting information

S1 Fig Boxplots for pain monitoring adherence. (PPTX)

S2 Fig Boxplots for sedation monitoring adherence. (PPTX)

S3 Fig Boxplots for delirium monitoring adherence. (PPTX)
