# Supplementary material for: Effects of pain, sedation and delirium monitoring on clinical and economic outcome: A retrospective study
Source: PLoS One. 2020 Sep 2;15(9):e0234801. doi: 10.1371/journal.pone.0234801 (PMC7467321; doi:10.1371/journal.pone.0234801)
Supplement: S2 Fig — (PPTX) [file pone.0234801.s002.pptx]

## Slide 1
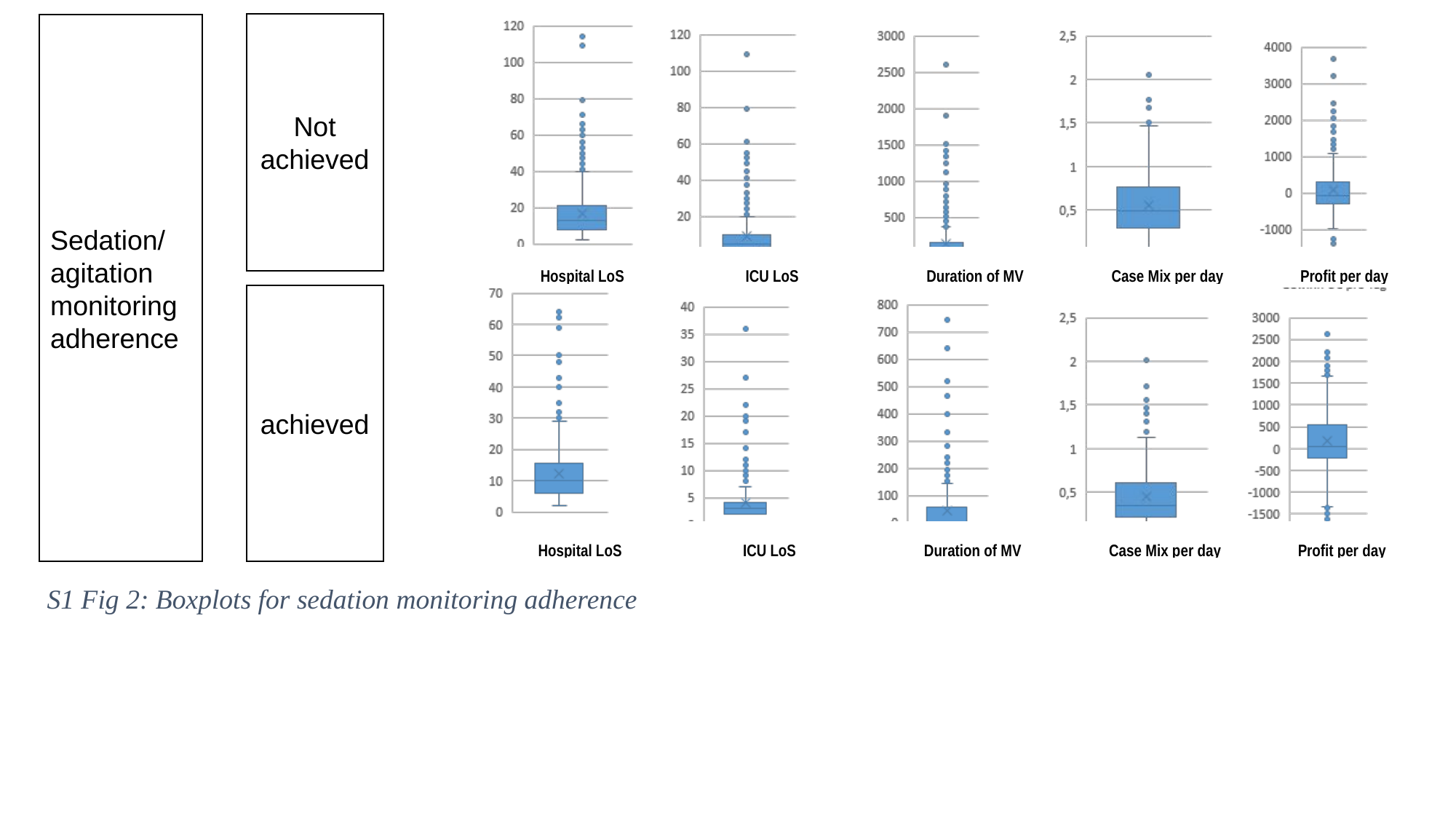

Not achieved
Sedation/
agitation monitoring adherence
| Hospital LoS | ICU LoS | Duration of MV | Case Mix per day | Profit per day |
| --- | --- | --- | --- | --- |
achieved
| Hospital LoS | ICU LoS | Duration of MV | Case Mix per day | Profit per day |
| --- | --- | --- | --- | --- |
S1 Fig 2: Boxplots for sedation monitoring adherence
